# Secret Remedies and Branded Products—IV

**Published:** 1908-03-28

**Authors:** 


### SECRET REMEDIES AND BRANDED PRODUCTS—IV.

[It is the object of these articles to show how departure from rational prescribing has fostered and encouraged a system which is open to many grievous abuses, of which self-medication is not the least. The writer appreciates the fact that many valuable preparations are sold under proprietary and protected names, and wishes it clearly to be understood that products which are recognised by medical practitioners on account of their real utility are in no way called in question.]

The Pure Food Act of the United States has been mentioned as having had a salutary influence on the nostrum evil in America, and although it may not be apparent at first sight how the law of a foreign land can in any way assist the campaign against secrecy in medicine in this country, it will do so very materially. The new law has, in fact, been the means of confirming many things that were dimly suspected, and of furnishing some very significant facts to work upon. It has been estimated that in America there are on the market more than 30,000 different proprietary medicines advertised directly to the public or sold through the intermediary of a physician's prescription, and the total annual sales have been calculated at 120,000,000 packages in one year.

These calculations were made prior to the enforcement of the Pure Food Act, since which time it is safe to infer from the following paragraph from an official report by Professor Wetherstroem that the traffic has suffered severely:—

"About a year ago there was a number of catarrh preparations brought to me for examination by the State, and one of them was a powder. It cost the retail druggists eight dollars a dozen, and, I suppose, was retailed to the public at a dollar a box. This box contained two powders, one white and one pink. I examined them and found nothing but sodium carbonate—common washing soda—worth at most two or three cents a pound. We could not do anything with the concern, which happened to be a reputable house, so we were very much pleased when the Federal law went into effect, and the first thing that was called to our attention was that this concern had discontinued sending out that preparation. The reasons were not given, but you can imagine that this preparation, being advertised for the cure of catarrh and containing what it did, they did not feel that they could disclose the name of the ingredients and sell it in the same way."

Now a very large proportion, probably more than one-third, of the secret remedies and branded medicinal products sold in this country are of American origin or manufacture, and although there are difficulties in the way of continuing the particular venture just referred to in America, there is, in this country, nothing to prevent this "reputable house" from selling for four shillings a halfpenny worth of washing soda in the guise of a catarrh cure, or to hinder the "reputable house" from warning the British public against "fraudulent imitations."

### An Infallible Cure for Morphinism.

An example of another class of secret medicine is also given in an official report of which the following is an extract:—

"Dr. Buckland had hit upon the only infallible cure for the morphine habit. His own portrait, beaming with benevolence, headed every advertisement. So kindly was the visage that it seemed to every sufferer like the face of an old friend. And such it proved to be, for after a few years some one found its exact duplicate in a dictionary of biography. There it was labelled 'Ludwig Spohr, German composer.' It was discovered at the same time that the morphine cure contained about half a grain of morphine hydrochloride to the ounce. No wonder the taking of the elixir satisfied the craving for the drug."

The same report contains the following statements:—

### The "Pure Vegetable" Fraud.

"Quite a number of pills are also advertised as being of an absolutely vegetable composition, although they contain mercury or one of its salts. By publishing that those pills are purely vegetable in their composition the proprietor caters for the trade of that class of people who are prejudiced against the use of calomel and other inorganic chemical medicinal agents. A good number of preparations claiming to contain popular substances, such as cod-liver oil, peptonate of iron, mandrake, arnica, cascara sagrada, cinchona, figs, and several others, have no traces of them, or if they contain them at all do so in considerably less quantity than that claimed."

Such falsely-described medicines cannot now be sold in America, but they can be and are being largely sold in Great Britain; in fact, one of the most extensively advertised nostrums in this country is a "purely vegetable" pill, which owes its medicinal effect—if any—to saltpetre.

### Old Drugs with New Names.

Just as the new regulations in America placed difficulties in the way of the proprietors of such quack compounds as those referred to, so it affected the manufacturer of many of those branded products which are directly advertised to the medical profession. In many cases these manufacturers wriggled out of the difficulty by completely altering the composition of their products without modifying in any way the claims that had previously been made for them. Some of these preparations, which were so changed in America, are widely prescribed by English physicians; but as regards the part of the output that is imported to this country the makers are under no obligation to change the composition, neither have they any compunction in continuing to mislead English prescribers as to the nature of the compound they induce them to prescribe. These preparations—and indeed nearly all the branded products and secret remedies offered to medical practitioners—are ordinary mixtures of well-known drugs, although it is often falsely claimed that they are new chemical compounds or contain new principles. If their true composition were known, prescribers would, in many cases, cease to use them, for sometimes these products contain the very drugs the physician wishes to avoid.

